# Supplementary material for: Poised PABP–RNA hubs implement signal-dependent mRNA decay in development
Source: Nat Struct Mol Biol. 2024 Jul 25;31(9):1439–47. doi: 10.1038/s41594-024-01363-x (PMC11402784; doi:10.1038/s41594-024-01363-x)
Supplement: Supplementary file 1 — Supplementary Fig. 1 [file 41594_2024_1363_MOESM1_ESM.pdf]

nature structural & molecular biology

Supplementary information https://doi.org/10.1038/s41594-024-01363-x

Poised PABP–RNA hubs implement signal-dependent mRNA decay in development

In the format provided by the authors and unedited

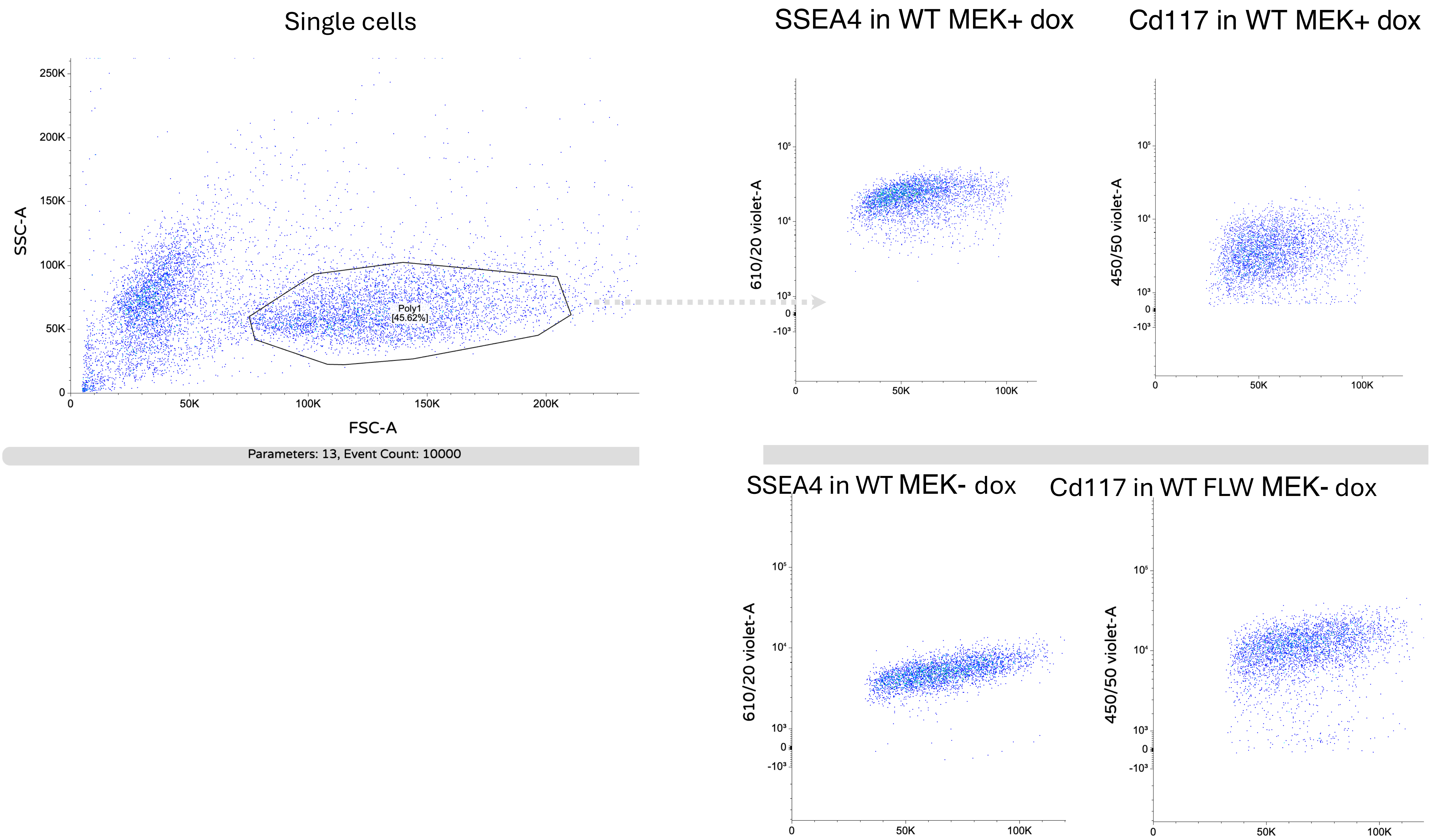
Single cells
SSC-A
250K
200K
150K
100K
50K
0
50K
100K
150K
200K
FSC-A
Poly1
[45.62%]
Parameters: 13, Event Count: 10000
SSEA4 in WT MEK+ dox
Cd117 in WT MEK+ dox
610/20 violet-A
450/50 violet-A
10^5
10^4
10^3
0
-10^3
0
50K
100K
SSEA4 in WT MEK- dox
Cd117 in WT FLW MEK- dox
