# Supplementary material for: Poised PABP–RNA hubs implement signal-dependent mRNA decay in development
Source: Nat Struct Mol Biol. 2024 Jul 25;31(9):1439–47. doi: 10.1038/s41594-024-01363-x (PMC11402784; doi:10.1038/s41594-024-01363-x)
Supplement: Supplementary file 2 — Reporting Summary [file 41594_2024_1363_MOESM2_ESM.pdf]

nature portfolio

Corresponding author(s): Jernej Ule and Miha Modic

Last updated by author(s): Apr 7, 2024

Reporting Summary

Nature Portfolio wishes to improve the reproducibility of the work that we publish. This form provides structure for consistency and transparency in reporting. For further information on Nature Portfolio policies, see our Editorial Policies and the Editorial Policy Checklist.

Statistics

For all statistical analyses, confirm that the following items are present in the figure legend, table legend, main text, or Methods section.

n/a	Confirmed	
☐	☒	The exact sample size (*n*) for each experimental group/condition, given as a discrete number and unit of measurement
☐	☒	A statement on whether measurements were taken from distinct samples or whether the same sample was measured repeatedly
☐	☒	The statistical test(s) used AND whether they are one- or two-sided<br>*Only common tests should be described solely by name; describe more complex techniques in the Methods section.*
☒	☐	A description of all covariates tested
☐	☒	A description of any assumptions or corrections, such as tests of normality and adjustment for multiple comparisons
☐	☒	A full description of the statistical parameters including central tendency (e.g. means) or other basic estimates (e.g. regression coefficient) AND variation (e.g. standard deviation) or associated estimates of uncertainty (e.g. confidence intervals)
☐	☒	For null hypothesis testing, the test statistic (e.g. *F*, *t*, *r*) with confidence intervals, effect sizes, degrees of freedom and *P* value noted<br>*Give P values as exact values whenever suitable.*
☒	☐	For Bayesian analysis, information on the choice of priors and Markov chain Monte Carlo settings
☒	☐	For hierarchical and complex designs, identification of the appropriate level for tests and full reporting of outcomes
☒	☐	Estimates of effect sizes (e.g. Cohen's *d*, Pearson's *r*), indicating how they were calculated

Our web collection on statistics for biologists contains articles on many of the points above.

Software and code

Policy information about availability of computer code

Data collection

Steady state super-resolution imaging of live ESCs and immunofluorescence images were acquired using a immunoOlympus IX83 microscope equipped with a VT-iSIM super resolution imaging system using MicroManager system. FACS data was acquired using a LSR Fortessa (BD Biosciences, BD FACSDiva Version 9.2.) flow cytometer. RT-qPCR data was acquired using QuantStudio7 (Thermo) and corresponding software. Western blot data was acquired using Amersham Imager 680 blot and gel imager. NGS libraries were sequenced as single end 100bp reads on Illumina HiSeq 4000. iCLIP experiments targeting LIN28A, PABPC1, and PABPC4 were sequenced on NovaSeq platform. Direct RNA sequencing for assessment of polyA-tail length was performed with MinION.

Data analysis

Data analysis, the software, and the settings used are described in "Methods". Code for bioinformatic analyses is available on GitHub (https://github.com/ulelab/LIN28A_RNPreassembly_bioinformatics), together with YAML environment files (containing versioned packages used for the analysis). Code and dependencies are also archived on Zenodo (https://zenodo.org/doi/10.5281/zenodo.10054297).
iCLIP-seq data was processed on iMaps Goodwright web-server (https://imaps.goodwright.com/), and the links to relevant collections are specified in Methods. The code and settings used in the iCLIP analysis pipeline (release v0.30) can be viewed at https://github.com/goodwright/imaps-nf, and are also archived on Zenodo (https://zenodo.org/doi/10.5281/zenodo.10054231).

Package versions used:
apeglm
bedtools (v2.29.2)
Clippy (v1.4.1)
Cutadapt (v3.4)
DESeq2 (v1.42)

fastp (v0.19.11)
Genome Analysis Toolkit (v3.5)
gbm (v2.1.8.1)
Guppy (v6.0.0)
iCount (v2.0.1.dev)
ImageJ (v1.52p)
MAJIQ (v2.1)
Integrative Genomics Viewer (v2.9.1)
minpack.lm (v1.2)
Nanopolish (v0.14.0)
Optuna (v3.1.0)
pybedtools (v0.9.0)
R (v4.0.3)
Salmon (v1.5.1 2)
samtools (v1.6)
seqkit (v2.3.1)
SHapley Additive exPlanations (SHAP) (v0.35.0)
STAR (v2.7.9a)
Snakemake (v5.3.0)
Slamdunk (v0.3.3)
TF-MoDISco Lite (v2.0.0)
VarScan (v2.4.1)
Pyranges (v0.0.117)

For manuscripts utilizing custom algorithms or software that are central to the research but not yet described in published literature, software must be made available to editors and reviewers. We strongly encourage code deposition in a community repository (e.g. GitHub). See the Nature Portfolio guidelines for submitting code & software for further information.

Data

Policy information about availability of data

All manuscripts must include a data availability statement. This statement should provide the following information, where applicable:

- Accession codes, unique identifiers, or web links for publicly available datasets
- A description of any restrictions on data availability
- For clinical datasets or third party data, please ensure that the statement adheres to our policy

Sequencing data related to iCLIP, Quantseq and Nanopore direct RNA sequencing experiments for Flag-tagged LIN28A-WT in 2iLIF and FGF2 treated cells, Flag-tagged LIN28A-S200A in FGF2 treated cells as well as for iCLIPs of PABPC1 and PABPC4 (in LIN28A KO cells with and without LIN28A overexpression) are available from ENA, with the accession code PRJEB60519. SlamSeq sequencing data, Quantseq data of LIN28A-GFP overexpression in LIN28A KO cells and LIN28A-GFP iCLIP experiments can be retrieved from GEO accession GSE169555.In addition, full data produced by iCLIP analysis pipeline for LIN28A-WT (in 2iL and FGF2 treated cells), LIN28A-S200A (in FGF2 treated cells) as well as for PABPC1 and PABPC4 (in LIN28A KO cells with and without LIN28A overexpression), can be accessed on the iMaps and Flow web-servers:
LIN28A iCLIP experiments: https://imaps.goodwright.com/collections/882635250203/; https://app.flow.bio/projects/882635250203/
PABPC iCLIP experiments: https://imaps.goodwright.com/collections/340215254997/; https://app.flow.bio/projects/340215254997/
To facilitate reproduction of our work, we archived key data from iCLIP analysis, used in downstream bioinformatic analyses–crosslink sites, peaks, and motif enrichments from PEKA–on Zenodo (https://zenodo.org/doi/10.5281/zenodo.10054231).

Research involving human participants, their data, or biological material

Policy information about studies with human participants or human data. See also policy information about sex, gender (identity/presentation), and sexual orientation and race, ethnicity and racism.

Reporting on sex and gender	Not applicable
Reporting on race, ethnicity, or other socially relevant groupings	Not applicable
Population characteristics	Not applicable
Recruitment	Not applicable
Ethics oversight	Not applicable

Note that full information on the approval of the study protocol must also be provided in the manuscript.

Field-specific reporting

Please select the one below that is the best fit for your research. If you are not sure, read the appropriate sections before making your selection.

☒ Life sciences ☐ Behavioural & social sciences ☐ Ecological, evolutionary & environmental sciences

For a reference copy of the document with all sections, see nature.com/documents/nr-reporting-summary-flat.pdf

Life sciences study design

All studies must disclose on these points even when the disclosure is negative.

Sample size	No statistical methods were used to predetermine the sample size. For RNA-seq on cell lines new to this study was determined by prior literature (for instance PMID: 34380047 and PMID: 28945705) and thereby we used similar experimental approaches rather than by power analysis.
Data exclusions	One iCLIP sample (LIN28A-S200A_ESC_LIF-CHIR-FGF0220626_MM_2) was excluded from subsequent analyses due to low read coverage.
Replication	The number of replicates used in each experiment are described in the figure legends and/or in the Methods section, as are the statistical tests used including the adjustment methods and alpha values for adjusted P values. All replicates successfully reproduced the presented findings, giving consistent results. LIN28A rescue was replicated in multiple independent cell lines, including multiple LIN28A KO clones were used. Statistical tests are selected appropriately to the analysed data, considering normality, variance, independence (paired or independent tests), and direction of effect (two-sided or one-sided tests). To compare two independent samples, with approximately normal distribution and approximately equal variance, we used two-sided two sample t-test; When the data is approximately normally distributed, but the equal variance criteria is not met, we used two-sided Welch's t-test; When the data did not meet the criteria for normality and/or equal variance, we used the two-sided Mann-Whitney-Wilcoxon rank-sum test. We occasionally employed the non-parametric two-sided Mann-Whitney-Wilcoxon rank-sum test in place of two sample t-test and Welch's t-test, due to fewer underying assumptions. In the figures, we indicate the comparisons of interest and report the exact P values when they are in range between 10e-4 to 10e-10; for P values lower than 10e-10, we label the P values as <10e-10.
Randomization	Randomization is not relevant to this study as no randomization is required due to the homogeneous nature of the cell lines. Furthermore, our RNA-seq discovery assays are high-throughput and were initially done in a hypotheses-free analysis. Observations in the RNA-seq were validated and confirmed by biochemical assays to bolster initial observations from the high-throughput assays.
Blinding	Immunoflourescence validation experiments were analysed blinded to genotype status.

Reporting for specific materials, systems and methods

We require information from authors about some types of materials, experimental systems and methods used in many studies. Here, indicate whether each material, system or method listed is relevant to your study. If you are not sure if a list item applies to your research, read the appropriate section before selecting a response.

Materials & experimental systems

n/a	Involved in the study	
☐	☒	Antibodies
☐	☒	Eukaryotic cell lines
☒	☐	Palaeontology and archaeology
☒	☐	Animals and other organisms
☒	☐	Clinical data
☒	☐	Dual use research of concern
☒	☐	Plants

Methods

n/a	Involved in the study	
☒	☐	ChIP-seq
☐	☒	Flow cytometry
☒	☐	MRI-based neuroimaging

Antibodies

Antibodies used

Antibodies used for cell immunoflourescence
LIN28A (A177 Cell Signaling and ab63740 Abcam)
Klf4 (AF3158, R&D Systems)
Nanog (8822, Cell Signaling)

Antibodies used for FACS experiments
SSEA1 (MC480, Thermo)
SSEA4 (MC813-70, Thermo)

Antibodies used for Western blotting
LIN28A (A177 Cell Signaling and AF3757, R&D Systems)
H3 (Abcam, ab1791)

GAPDH (Cell Signaling, 2118S)

Antibodies used for iCLIP
GFP polyclonal Antibody - Thermo Fisher A6455
LIN28A polyclonal Antibody - Cell signaling #3978
PABPC1 - Abcam ab21060 and Proteintech 10970-1-AP
PABPC4 - Proteintech 14960-1-AP

Validation

Antibodies used for Western blotting and cell immunoflourescence:
LIN28A (A177 Cell Signaling). An antibody routinely used as a first choice for LIN28A immunoprecipitations, Western Blots and immunoflourescence: Product website outlines 42 relevant citations (https://www.cellsignal.co.uk/products/primary-antibodies/lin28a-a177-antibody/3978?N=0+4294956287&Nrpp=200&No=3200&fromPage=plp), including publications that validated the specificity of A177 LIN28A Ab when compared to a cell line not expressing LIN28A (PMID: 27992407).

LIN28A (ab63740 Abcam). Relevant newer monoclonal antibody that has already been cited 8times and we have thoughtfully validated it's specificity for Immunofluorescence and WB using a LIN28A KO cell line (Extended Data Figure 1). Product Website: https://www.abcam.com/lin28-antibody-ab63740.html

Klf4 (AF3158, R&D Systems). Widely used antibody that has been extensively validated (for instance PMID: 30540935) and published with 33 citations according to the product website (https://www.rndsystems.com/products/mouse-klf4-antibody_af3158#product-citations)

Nanog (8822, Cell Signaling). Widely used antibody that has been widely accepted as a marker of naive pluripoteny stem cells, is extensively validated (e.g. PMID: 32034125) and published with 32 citations according to the product website (https://www.cellsignal.com/products/primary-antibodies/nanog-d2a3-xp-rabbit-mab-mouse-specific/8822)

Two PABPC1 antibodies were cross-validated (ab21060 and 10970-1-AP) with comparable enrichment in kmers and peak-calling. Antibodies have also been used in 120 and 4 citations respectively.

PABPC4 antibody (Proteintech) was validated using a KO cell lines and cited in 6 additional publications. https://www.ptglab.com/products/PABPC4-Antibody-14960-1-AP.htm

The following secondary antibodies were used that were previously tested in PMID: 31047794:
Donkey anti-rabbit IgG 555 Invitrogen Cat no. A31572; RRID: AB_162543
Donkey anti-goat IgG 488 Invitrogen Cat no. A11055; RRID: AB_2534102

Antibodies used for FACS experiments:
All listed antibodies have already been thoroughly tested and validated with isotype controls for the same application of detecting cell-Surface Markers Specific to Naive and Formative/Primed Pluripotent States (PMID: 31078527 and PMID: 31047794)
Pacific Blue™ anti-mouse CD117 (c-Kit) Antibody (105820, Biolegen)
SSEA4 Monoclonal Antibody (MC-813-70, Thermo)

Eukaryotic cell lines

Policy information about cell lines and Sex and Gender in Research

Cell line source(s)

IDG3.2. | Helmholtz Zentrum Munich Transgenic Facility (AG Schirge)
V6.5. | Novus Bio and Harvard (gift of Prof. George Daley).
All CRISPR and other genetic modifications were perfomed on outlined cell lines by paper authors.

Authentication

CRISPR modifications were genotyped and assessed by Western Blot and PCR.

Mycoplasma contamination

All cell lines were tested free of mycoplasma contamination by Cell Services of the Francis Crick Institute, London, UK.

Commonly misidentified lines
(See ICLAC register)

No commonly misidentified lines were used in this study.

Flow Cytometry

Plots

Confirm that:

☒ The axis labels state the marker and fluorochrome used (e.g. CD4-FITC).

☒ The axis scales are clearly visible. Include numbers along axes only for bottom left plot of group (a 'group' is an analysis of identical markers).

☒ All plots are contour plots with outliers or pseudocolor plots.

☒ A numerical value for number of cells or percentage (with statistics) is provided.

Methodology

Sample preparation

For flow cytometry experiments, single-cell suspensions were made using Accutase (A6964, Sigma-Aldrich) for 5 min in 37 °C; or Enzyme-Free Cell Dissociation Buffer (13151014, Gibco) for 30 min at 37 °C and washed with 5% FBS (EmbryoMax® ES Cell

	Qualified FBS, ES-009-B, Merck) in PBS, incubated with fluorophore-conjugated antibodies for 30-60 min on ice. Cells were centrifuged, resuspended and 10000 cells was analyzed using a LSR Fortessa cytometers (BD Biosciences, BD FACSDiva Version 9.2.). Cell debris were excluded by forward and side scatter gating.
Instrument	LSR Fortessa (BD Biosciences, BD FACSDiva Version 9.2.).
Software	Data was acquired using BD FACSDiva and analyzed using FlowJo V10.6.1.
Cell population abundance	No sorting was performed. The entire cell population was analysed. No post-gating sort was performed.
Gating strategy	Forward and side scatter were set as to gate single cells.

☒ Tick this box to confirm that a figure exemplifying the gating strategy is provided in the Supplementary Information.
