# Supplementary figures and images for: Poised PABP–RNA hubs implement signal-dependent mRNA decay in development
Source: Nat Struct Mol Biol. 2024 Jul 25;31(9):1439–47. doi: 10.1038/s41594-024-01363-x (PMC11402784; doi:10.1038/s41594-024-01363-x)

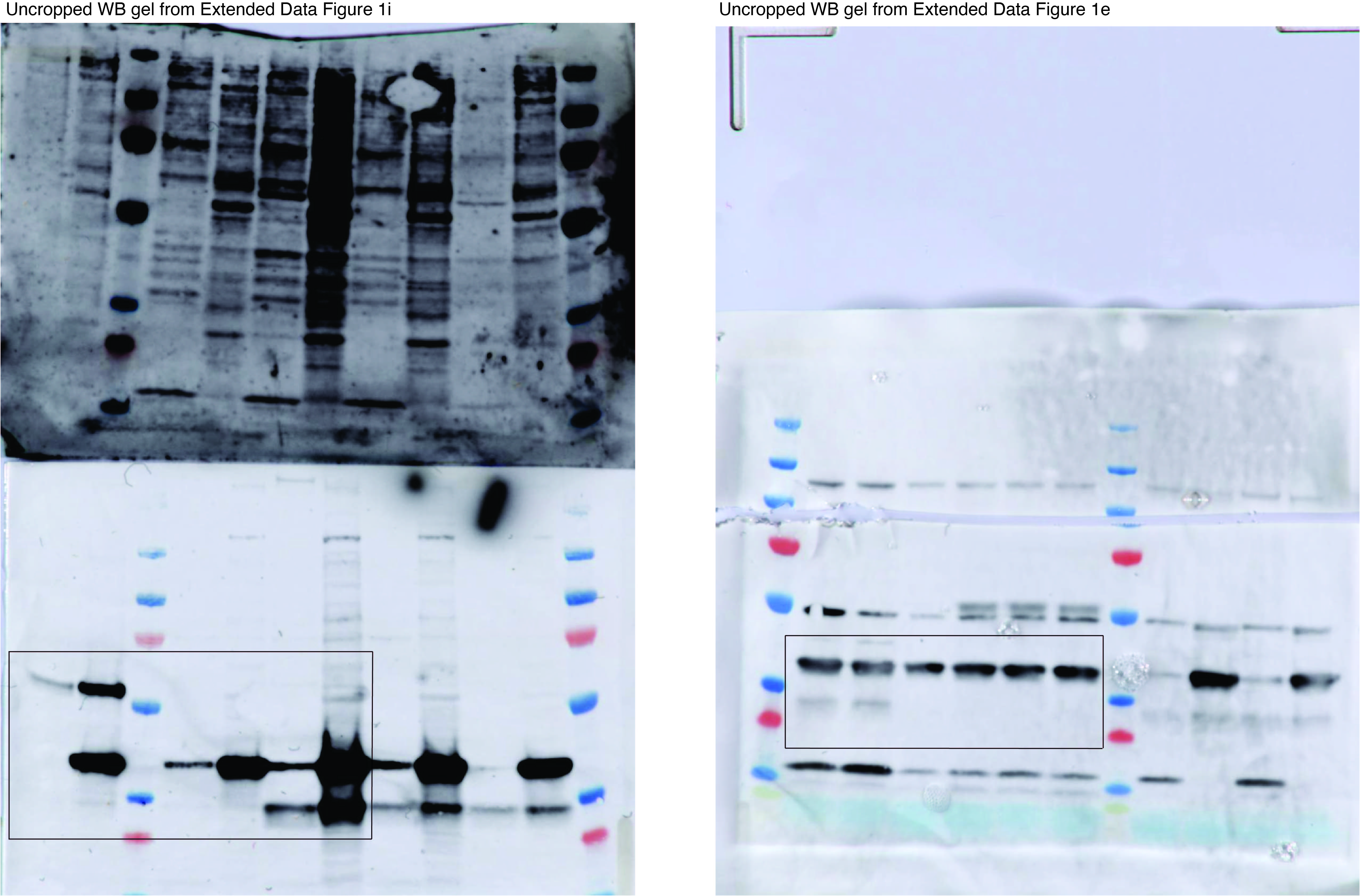

Supplement: Supplementary file 16 — Uncropped western blot gels. [file 41594_2024_1363_MOESM16_ESM.jpg]

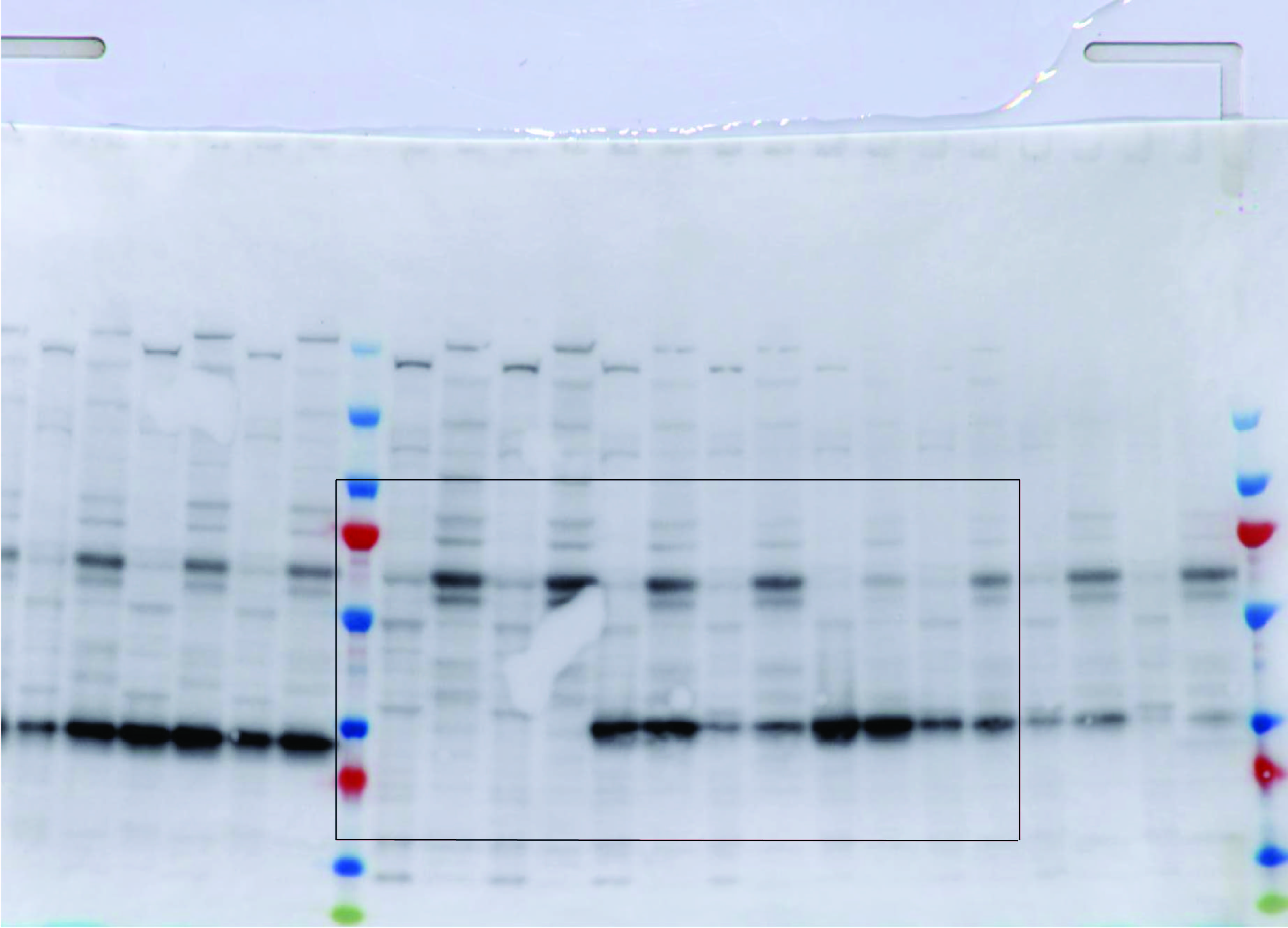

Supplement: Supplementary file 17 — Uncropped western blot gels. [file 41594_2024_1363_MOESM17_ESM.jpg]
